# Prediction Models for Bronchopulmonary Dysplasia in Preterm Infants: A Systematic Review

**DOI:** 10.3389/fped.2022.856159

**Published:** 2022-05-12

**Authors:** Hai-Bo Peng, Yuan-Li Zhan, You Chen, Zhen-Chao Jin, Fang Liu, Bo Wang, Zhang-Bin Yu

**Affiliations:** ^1^Department of Neonatology, Affiliated Shenzhen Baoan Women’s and Children’s Hospital, Jinan University, Shenzhen, China; ^2^Department of Pediatrics, The Affiliated Suqian First People’s Hospital of Nanjing Medical University, Suqian, China; ^3^Department of Neonatology, Shenzhen People’s Hospital, The Second Clinical Medical College, Jinan University, Shenzhen, China; ^4^The First Affiliated Hospital, Southern University of Science and Technology, Shenzhen, China

**Keywords:** prediction, model, bronchopulmonary dysplasia, preterm infants, systematic review

## Abstract

**Objective:**

To provide an overview and critical appraisal of prediction models for bronchopulmonary dysplasia (BPD) in preterm infants.

**Methods:**

We searched PubMed, Embase, and the Cochrane Library to identify relevant studies (up to November 2021). We included studies that reported prediction model development and/or validation of BPD in preterm infants born at ≤32 weeks and/or ≤1,500 g birth weight. We extracted the data independently based on the CHecklist for critical Appraisal and data extraction for systematic Reviews of prediction Modelling Studies (CHARMS). We assessed risk of bias and applicability independently using the Prediction model Risk Of Bias ASsessment Tool (PROBAST).

**Results:**

Twenty-one prediction models from 13 studies reporting on model development and 21 models from 10 studies reporting on external validation were included. Oxygen dependency at 36 weeks’ postmenstrual age was the most frequently reported outcome in both development studies (71%) and validation studies (81%). The most frequently used predictors in the models were birth weight (67%), gestational age (62%), and sex (52%). Nearly all included studies had high risk of bias, most often due to inadequate analysis. Small sample sizes and insufficient event patients were common in both study types. Missing data were often not reported or were discarded. Most studies reported on the models’ discrimination, while calibration was seldom assessed (development, 19%; validation, 10%). Internal validation was lacking in 69% of development studies.

**Conclusion:**

The included studies had many methodological shortcomings. Future work should focus on following the recommended approaches for developing and validating BPD prediction models.

## Introduction

Preterm infant survival has increased in the last three decades (1–3), while bronchopulmonary dysplasia (BPD) remains the most prevalent serious complication of prematurity, affecting 10.8–37.1% of preterm neonates born at 240/7 to 316/7 weeks’ gestational age and birth weight <1,500 g (4). As survivors with BPD have high risk of poor long-term pulmonary and neurodevelopmental outcomes in childhood and even adulthood (5–8), it is imperative to optimize BPD prevention and treatment strategies. Early identification of infants at risk of developing BPD would benefit preventive interventions when airway injury is still functional and reversible. To aid health care providers in estimating the probability of BPD occurrence in the future and to inform decision-making, many models for predicting BPD have been established in recent years. Nevertheless, such models are often of variable quality and yield inconsistent findings, leading to confusion or uncertainty among health care providers regarding which model to use or recommend.

In a 2013 systematic review, Onland et al. reported 26 prediction models for assessing the probability of BPD or death in all preterm infants born at <37 weeks’ gestation, where most existing clinical prediction models were poor to moderate BPD predictors (9). Furthermore, during that review, no guides for systematic reviews of prediction modeling studies or standardization tools for assessing the prediction models’ risk of bias (ROB) were available. Since then, more BPD prediction modeling studies have been published, whereas systematic reviews of such studies have not yet been updated in the last 9 years. The guideline CHecklist for critical Appraisal and data extraction for systematic Reviews of prediction Modelling Studies (CHARMS) has been available since 2014 (10), and the Prediction model Risk Of Bias ASsessment Tool (PROBAST) for assessing the ROB and applicability of prediction model studies has been available since 2019 (11).

Accordingly, the present systematic review was aimed at updating the systematic review of BPD prediction models and critically evaluating the methods and reporting of studies that developed or externally validated prediction models for BPD in preterm infants born at ≤32 weeks and/or ≤1,500 g birth weight based on the CHARMS checklist and PROBAST.

## Methods

This systematic review of all studies on prediction models for BPD in preterm infants is reported according to Preferred Reporting Items for Systematic Reviews and Meta-Analyses (PRISMA) guidelines (12).

### Search Strategy

PubMed (MEDLINE), Embase, and the Cochrane Library were systematically searched from inception through to 12 November 2021, for studies reporting prediction models of BPD in preterm infants. We identified relevant studies and maximized search accuracy using the following terms: BPD, chronic lung disease, preterm infants, and prediction. The online Supplementary Material 1 shows the electronic search strategies. The search was not limited by language.

### Eligibility Criteria

Articles were included if: (1) the target population was preterm infants born at ≤32 weeks and/or ≤1,500 g birth weight; (2) the study detailed prediction model development and/or external validation; (3) the main prediction outcome was BPD, defined as oxygen requirement at 28 days of life (BPD28) and/or oxygen requirement at 36 weeks’ postmenstrual age (PMA) (BPD36); (4) the model was constructed with at least two predictors; and (5) the purpose of the model was for predicting BPD in preterm infants from the first 2 weeks of life. Articles were ineligible when the studies used the data of infants born before 1990, as surfactant was not routinely used before this year (pre-surfactant era); if the outcome to be predicted was the composite outcome “BPD or death”; when the prognostic use of lung ultrasound scores (LUS) was investigated; when the study was conducted at high altitudes; when it was only a methodological study; when the article was not published in English; or when the article was a conference abstract, review, or letter.

### Study Selection and Data Extraction

Two reviewers independently screened the titles, abstracts, and full texts in duplicate for eligibility. In case of discrepancies, a third reviewer was involved to establish consensus. The reviewers used a standardized data extraction form based on the CHARMS checklist (10). The following items were extracted from the studies on prediction model development: study design, study population, predicted outcome and time horizon, intended moment of model use, number of candidate predictors, sample size, number of events, missing data approach, variables selection method, modeling method, model presentation, predictors included in the final model, internal validation method, and assessment of model performance (i.e., discrimination and calibration). The following items were extracted from the prediction model external validation studies: study design, study population, predicted outcome and time horizon, intended moment of model use, sample size, number of events, missing data approach, and assessment of model performance (i.e., discrimination and calibration). The events per variable (EPV) was defined as the number of events divided by the number of candidate predictor variables used. The outcome BPD28 was defined as oxygen dependency at 28 days of life; BPD36 was defined as oxygen dependency at 36 weeks PMA.

### Assessment of Bias

We assessed the ROB and applicability of each article with PROBAST. PROBAST consists of 20 signaling questions across four domains (participants, predictors, outcome, and analysis). The ROB and applicability of original studies were classified as high, low, or unclear for each domain *via* comprehensive evaluation. Only if each domain had low ROB would a study be classified as overall low ROB.

### Model Performance

The results of the development and external validation studies were summarized by using descriptive statistics. If an article described the development or external validation of multiple (existing) models, separate data extraction for each model was conducted. Each model’s predictive performance, including model discrimination and calibration measures, was extracted. Discrimination is often quantified by the C statistic. The C statistic is the most commonly used measure for determining the discriminative performance for binary outcomes. Generally, a C statistic < 0.6 is considered poor, a C statistic between 0.6 and 0.75 is possibly helpful, a C statistic > 0.75 is clearly useful (13). Calibration is often quantified by the calibration intercept and calibration slope.

## Results

**FIGURE 1:**
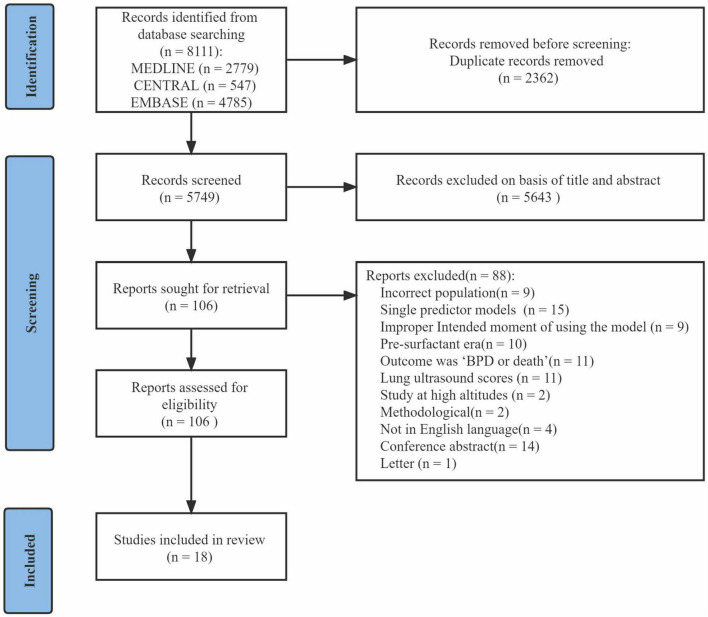
Preferred Reporting Items for Systematic Reviews and Meta-Analyses (PRISMA) flow diagram.

After excluding duplicates, the initial search returned 5,749 articles. After title and abstract screening, 106 articles were provisionally selected for full-text screening. Subsequently, 88 articles were excluded, among which 11 articles used the composite outcome “BPD or death.” In total, 18 studies (14–31) were included in this systematic review (Figure 1). Eight studies (14, 16, 19, 21, 22, 25–27) described model development without external validation, five studies (15, 17, 24, 29, 30) described model development with external validation in independent data, and five studies (18, 20, 23, 28, 31) described external validation with or without model updating.

### Characteristics of Studies Describing Bronchopulmonary Dysplasia Prediction Model Development

Thirteen studies described BPD prediction model development, in which 21 models were developed. Table 1 shows the key characteristics of study design, study population, outcome, and intended moment of model use in the included model development studies. Table 2 shows the study and performance characteristics of the developed models.

**TABLE 1:** Design characteristics of the 13 studies describing the development of BPD prediction models.

Study	Country	Study design	Years of data	Study population	Timing of BPD	Intended moment of model use	Models, *n*	Differences between models caused by differences in the following:
El Faleh et al. (17)	Switzerland	Registry	2009–2010	BW < 1,501 g and/or GA between 23 0/7 and 31 6/7 weeks	DOL28 and PMA36	1 day	2	Timing of BPD
Shim et al. (15)	Korea	Registry	2013–2016	BW < 1,500 g and GA ≥ 22 weeks	PMA36	1 h	3	Severity of BPD
Ushida et al. (14)	Japan	Registry	2006–2015	GA < 32 weeks and BW ≤ 1,500 g	PMA36	At birth	1	NA
Verder et al. (16)	Denmark	Prospective cohort	2019	GA 24–31 weeks	DOL28	At birth	1	NA
Valenzuela-Stutman et al. (19)	Argentina, Chile, Paraguay, Peru, Uruguay	Registry	2001–2015	BW 500–1,500 g	PMA36	At birth, 3 day, 7 day, 14 day	4	Intended moment of using the model
Sullivan et al. (21)	United States	Prospective cohort	2009–2015	BW < 1,500 g	PMA36	7 day	3	Predictors
Bentsen et al. (22)	United States	Prospective cohort	2014–2016	GA < 28 weeks	PMA36	2 day	1	NA
Gursoy et al. (24)	Turkey	Retrospective cohort	2006–2009	GA ≤ 32 weeks and BW ≤ 1,500 g	DOL28	3 day	1	NA
Tian et al. (26)	China	Prospective cohort	2010–2011	BW ≤ 1,500 g and GA ≤ 32 weeks	DOL28	At birth	1	NA
Wang et al. (25)	China	Prospective cohort	2011–2013	BW ≤ 1,500 g and GA ≤ 32 weeks	DOL28	14 day	1	NA
May et al. (27)	United Kingdom	Prospective cohort	2004–2007	GA 24–32 weeks	DOL28	14 day	1	NA
Henderson-Smart et al. (29)	Australia, New Zealand	Registry	1998–1999	GA 22–31 weeks	PMA36	At birth	1	NA
Kim et al. (30)	Korea	Retrospective cohort	1997–1999	BW < 1,500 g	PMA36	4 day, 7 day, 10 day	1	NA

*BPD, bronchopulmonary dysplasia; BW, birth weight; DOL, days of life; GA, gestational age; PMA, postmenstrual age; NA, not applicable.*

**TABLE 2:** Study and performance characteristics of the developed prediction models.

Study	Outcome	Intended moment of model use	Sample size	Events	EPV	Missing data	Univariable analysis	Modeling method	Model presentation	Predictors, n	Discrimination (C statistic)	Calibration	Internal validation
El Faleh et al. (17)	BPD28	1 day	1,232	266	22.2	Complete case study	Yes	Logistic regression	Formula; web calculator	7	0.88	HL test	NR
	BPD36	1 day	1,225	138	11.5	Complete case study	Yes	Logistic regression	Formula	5	0.84	HL test	NR
Shim et al. (15)	All BPD36	1 h	4,600	2,583	184.5	Complete case study	No	Logistic regression	Formula	4	0.908 (0.899–0.916)	NR	NR
	Moderate to severe BPD36	1 h	4,600	1,370	97.9	Complete case study	No	Logistic regression	Formula	7	0.815 (0.802–0.828)	NR	NR
	Severe BPD36	1 h	4,600	818	58.4	Complete case study	No	Logistic regression	Formula	8	0.815 (0.800–0.831)	NR	NR
Ushida et al. (14)	BPD36	At birth	18,858	4,986	415.5	Complete case study	No	Logistic regression	Formula	8	0.80 (0.79–0.81)	Calibration plots	Split-sample validation
Verder et al. (16)	BPD28	At birth	61	26	2	NR	No	SVM	NR	4	NR	NR	Cross validation
Valenzuela-Stutman et al. (19)	BPD36	At birth	16,407	2,580	215	NR	No	Logistic regression	Web calculator	5	0.788	NR	Split-sample validation
	BPD36	3 day	16,407	2,580	151.8	NR	No	Logistic regression	Web calculator	5	0.818	NR	Split-sample validation
	BPD36	7 day	16,407	2,580	143.3	NR	No	Logistic regression	Web calculator	5	0.827	NR	Split-sample validation
	BPD36	14 day	16,407	2,580	127.5	NR	No	Logistic regression	Web calculator	5	0.894	NR	Split-sample validation
Sullivan et al. (21)	BPD36	7 day	443	159	NA	Complete case study	No	Logistic regression	NR	6	0.921 (0.897–0.945)	NR	Bootstrapping
	BPD36	7 day	443	159	NA	Complete case study	No	Logistic regression	NR	5	0.886 (0.854–0.913)	NR	Bootstrapping
	BPD36	7 day	443	159	NA	Complete case study	No	Logistic regression	NR	11	0.935 (0.920–0.951)	NR	Bootstrapping
Bentsen et al. (22)	BPD36	2 day	37	18	1.1	NR	Yes	Logistic regression	NR	3	0.893 (0.735–0.973)	NR	NR
Gursoy et al. (24)	BPD28	3 day	652	150	4.8	NR	Yes	Logistic regression	Scoring system	7	0.93	NR	NR
Tian et al. (26)	BPD28	14 day	73	24	NA	Complete case study	Yes	Logistic regression	NR	2	0.974	NR	NR
Wang et al. (25)	BPD28	At birth	134	35	NA	NR	No	Logistic regression	NR	2	0.849	NR	NR
May et al. (27)	BPD28	14 day	78	39	2.6	NR	No	Logistic regression	NR	2	0.97	NR	NR
Henderson-Smart et al. (29)	BPD36	At birth	5,599	1,235	58.8	Complete case study	Yes	Logistic regression	NR	3	0.84	HL test	NR
Kim et al. (30)	BPD36	4 day	161	30	1.6	NR	No	Logistic regression	Scoring system	8	0.76	NR	NR

*BPD, bronchopulmonary dysplasia; DOL, days of life; EPV, events per variable; HL test, Hosmer–Lemeshow test; NA, not available; NR, not reported; PMA, postmenstrual age; SVM, support vector machine.*

#### Study Design

Eleven included studies (85%) originated from registry or prospective cohorts; two studies (15%) were derived from retrospective cohorts. The data used for developing the models were collected between 1997 and 2019. Of all 13 model development studies, four (31%) used only gestational age as the inclusion criterion, three studies (23%) used only birth weight as the inclusion criterion, and six studies (46%) used both gestational age and birth weight as inclusion criteria. All studies were developed based on statistical methods. Twelve studies (92%) used logistic regression as the prediction modeling approach; one study (8%) used machine learning.

#### Outcome to Be Predicted

The outcome to be predicted in all included studies was BPD, yet the definitions of BPD varied across the models. Six models (29%) used BPD28 as the primary outcome; the median incidence was 29% (range, 22–50%). Fifteen models (71%) used BPD36 as the primary outcome, with values of 11–56% (median, 22%). Eighteen models (86%) were developed to predict the risk of developing BPD within 7 days of life, and three models (14%) were developed to be used between 7 and 14 days of life.

#### Predictors

Ten of the 13 studies reported the number of candidate predictors considered for inclusion in the BPD prediction models, with 12–31 candidate predictors (median, 15). Two to 11 predictors were included in the final model (median, 5). Five studies (38%) used univariable analysis to select predictors in the multivariable analyses.

**FIGURE 2:**
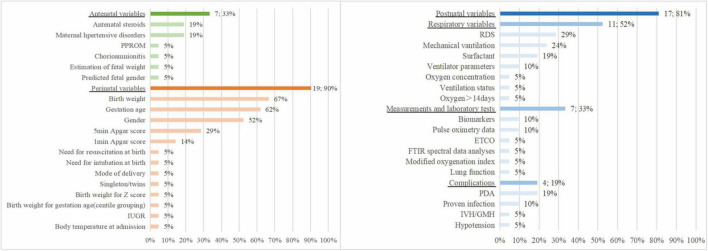
Predictors included in the final development models.

Figure 2 shows the predictors included in the final prediction models. Nineteen models (90%) used perinatal variables, 7 studies (33%) used antenatal variables, and 17 models (81%) used postnatal variables. The most frequently included predictor in the 21 prediction models was birth weight (*n* = 14, 67%), followed by gestational age (*n* = 13, 62%), sex (*n* = 11, 52%), 5-min Apgar score (*n* = 6, 29%), respiratory distress syndrome (*n* = 6, 29%), mechanical ventilation (*n* = 5, 24%), antenatal steroids (*n* = 4, 19%), maternal hypertensive disorders (*n* = 4, 19%), surfactant (*n* = 4, 19%), and patent ductus arteriosus (*n* = 4, 19%).

#### Sample Size

The models were developed with 37–18,858 participants (median, 1,225), and there were 18–4,986 events (median, 159). The EPV could be calculated in 16 models (76%) with a median of 59 and a range of 1–416. The EPV was <10 in 31% of the models in which it was calculated.

#### Missing Data

Seven studies (54%) did not mention missing data. Six studies (46%) mentioned the methods for addressing missing data, where they all used complete case analysis.

#### Model Presentation

Presentation was available for 12 models (57%). Five models were presented as regression formulae, two models were presented as scoring systems, four models were presented as web calculators, and one model was presented as both a regression formula and web calculator.

#### Apparent Predictive Performance

Twelve studies (95%) assessed discrimination with the C statistic, with values of 0.76–0.97. Calibration was assessed for four models (19%), two models used the Hosmer–Lemeshow goodness-of-fit test, and one model used calibration plots.

#### Internal Validation

Nine studies (69%) did not report internal validation of the developed models. Nine models developed in four studies were internally validated. Validation was performed for five models (56%) with split sampling, in one model (11%) with cross-validation, and in three models (33%) with bootstrapping.

#### Risk of Bias and Applicability Assessment of the Included Model Development Studies

**FIGURE 3:**
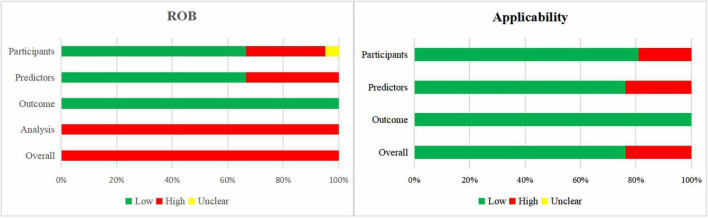
Risk of bias and applicability assessment of developed models using Prediction model Risk Of Bias ASsessment Tool (PROBAST).

Figure 3 shows a summary of the ROB and applicability for all developed models. For the domain outcome, the ROB of all models was considered low, as a broad definition of BPD was accepted. There was high participants’ domain-related ROB in 29% of the models. For the domain predictors, 33 and 67% of the models had high and low ROB, respectively. The domain analysis was assessed as having high ROB in all prediction models. No study handled missing data appropriately, as information on missing data was rarely reported or participants with missing data were omitted. Prediction model calibration was insufficient, as only one study reported calibration plots, while the other studies did not report calibration or only used the Hosmer–Lemeshow test. In summary, the overall ROB was high across all models.

When the 21 models were assessed according to applicability concerns, 24% of the models were assessed as high concern due to the inclusion of participants different from those in our research question (*n* = 4) or inconsistency between predictors and the review question (*n* = 5).

### Characteristics of Studies Describing External Validation of the Bronchopulmonary Dysplasia Prediction Models

We included 10 studies that externally validated 21 BPD prediction models (Table 3). Five of these studies also described prediction model development. Table 4 shows the study and performance characteristics of the validated models.

**TABLE 3:** Design characteristics of the 10 studies describing external validation of BPD prediction models.

Study	Country	Study design	Years of data	Study population	Timing of BPD	Intended moment of model use	Model validated
El Faleh et al. (17)	Switzerland	Registry	2014–2015	BW < 1,501 g and/or GA between 23 0/7 and 31 6/7 weeks	DOL28 or PMA36	1 day	El Faleh et al. (17)
Shim et al. (15)	Korea	Registry	2017	BW < 1,500 g and GA ≥ 22 weeks	PMA36	1 h	Shim et al. (15)
Bhattacharjee et al. (18)	United States	Retrospective cohort	2012–2013	BW < 1,500 g	PMA36	3 day	RSS
Lee et al. (20)	Korea	Registry	2013–2016	BW < 1,500 g and GA 22–32 weeks	PMA36	1 h	CRIB II; CRIB II-BE
Sullivan et al. (23)	United States	Retrospective cohort	2004–2014	BW < 1,500 g	PMA36	12 h, 1 day, 7 day	aHRC-24h, aHRC-7d, SNAP-II, CRIB-II
Gursoy et al. (24)	Turkey	Prospective cohort	2012	GA ≤ 32 weeks and BW ≤ 1,500 g	DOL28	3 day	BPD-TM score
May et al. (28)	United Kingdom	Retrospective cohort	1995–1998	BW < 1,500 g and GA < 33 weeks	DOL28 or PMA36	2 day	Simple pulmonary score
Henderson-Smart et al. (29)	Australia, New Zealand	Registry	2000–2001	GA 22–31 weeks	PMA36	At birth	Henderson-Smart et al. (29)
Kim et al. (30)	Korea	Prospective cohort	2000–2001	BW < 1,500 g	PMA36	4 day, 7 day, 10 day	SMUMRV; Yoder model (55)
Chien et al. (31)	Canada	Registry	1996–1997	GA ≤ 32 weeks	PMA36	12 h	SNAP-II + GA, SGA, sex, low Apgar score, and outborn status

*BPD, bronchopulmonary dysplasia; BPD-TM, bronchopulmonary dysplasia test measure; BW, birth weight; CRIB, Clinical Risk Indicator fores; CRIB II-BE, Clinical Risk Indicator fores omitting base excess; DOL, days of life; GA, gestational age; HRC, heart rate characteristics; aHRC-24h, average first day HRC index; aHRC-7d, average first week HRC index within 7 days of birth; PMA, postmenstrual age; RSS, respiratory severity score; SGA, small for gestational age; SMUMRV, modified respiratory variables; SNAP-II, Score for Neonatal Acute Physiology-II.*

**TABLE 4:** Study and performance characteristics of externally validated models.

Study	Model	Outcome	Sample size	Outcome events	Missing data	Discrimination (C-statistic)	Calibration
El Faleh et al. (17)	El Faleh et al. (17)	BPD28	1,733	437	Complete case study	0.92	NR
	El Faleh et al. (17)	BPD36	1,724	191	Complete case study	0.88	NR
Shim et al. (15)	Shim et al. (15)	BPD36 (all grade)	1,740	1,003	Complete case study	NR	NR
	Shim et al. (15)	BPD36 (moderate to severe)	1,740	563	Complete case study	NR	NR
	Shim et al. (15)	BPD36 (severe)	1,740	388	Complete case study	NR	NR
Bhattacharjee et al. (18)	RSS	BPD36	69	31	NR	0.61	NR
Lee et al. (20)	CRIB II	BPD36	4,694	1,443	Complete case study	0.77 (0.76–0.79)	NR
	CRIB II-BE	BPD36	6,038	1,916	Complete case study	0.77 (0.76–0.78)	NR
Sullivan et al. (23)	aHRC-24h	BPD36	566	98	NR	0.827	NR
	aHRC-7d	BPD36	566	98	NR	0.827	NR
	SNAP-II	BPD36	566	98	NR	0.839	NR
	CRIB-II	BPD36	566	98	NR	0.840	NR
Gursoy et al. (24)	BPD-TM score	BPD28	172	54	NR	0.903	NR
May et al. (28)	Simple pulmonary score-day 2	BPD28	75	32	NR	0.79 (cohort 1), 0.84 (cohort 2)	NR
	Simple pulmonary score-day 2	BPD36	75	22	NR	0.86 (cohort 1), 0.76 (cohort 2)	NR
	Simple pulmonary score-day 7	BPD28	75	32	NR	0.75 (cohort 1), 0.97 (cohort 2)	NR
	Simple pulmonary score-day 7	BPD36	75	22	NR	0.83 (cohort 1), 0.88 (cohort 2)	NR
Henderson-Smart et al. (29)	Henderson-Smart et al. (29)	BPD36	5,854	1,475	Complete case study	0.84	HL test
Kim et al. (30)	SMUMRV	BPD36	96	9	NR	0.90–0.94	NR
	Yoder model (55, 57)	BPD36	96	9	NR	0.92–0.96	NR
Chien et al. (31)	SNAP-II + GA, SGA, sex, low Apgar, and outborn status	BPD36	4226	NR	Complete case study	0.83	HL test

*BPD, bronchopulmonary dysplasia; BPD-TM, bronchopulmonary dysplasia test measure; BW, birth weight; CRIB, Clinical Risk Indicator fores; CRIB II-BE, Clinical Risk Indicator fores omitting base excess; DOL, days of life; GA, gestational age; HL test, Hosmer–Lemeshow test; HRC, heart rate characteristics; NR, not reported; PMA, postmenstrual age; RSS, respiratory severity score; SGA, small for gestational age; SMUMRV, modified respiratory variables; SNAP-II, Score for Neonatal Acute Physiology-II.*

#### Models Validated

The most frequently validated models were CRIB-II (Clinical Risk Indicator fores-II) and SNAP-II (Score for Neonatal Acute Physiology-II); both were externally validated twice. The other models were externally validated once.

#### Study Design

Eight validation studies (80%) used existing data to externally validate a BPD prediction model. Two studies (20%) collected prospective data for external validation. The data used for validating the BPD prediction models were all collected between 1995 and 2017.

#### Outcome

Four models (19%) used BPD28 as the outcome. The incidence of BPD28 was 25–43% (median, 37%). Seventeen models (81%) used BPD36 as the outcome. The incidence of BPD36 was 9–58% (median, 24%).

#### Sample Size

All studies reported the number of patients. The number of event patients could be identified in nine studies (90%). The validation articles included 69–6,038 patients (median, 566). The median number of event patients was 98 (range, 9–1,916). Twelve models (60%) had <100 event patients.

#### Missing Data

Five studies mentioned missing data (50%). These studies all used complete case analysis to address the missing data.

#### Predictive Performance

Nine of the 10 validation studies (90%) assessed model discrimination with the C statistic (range, 0.61–0.97). Two models (10%) reported model calibration using the Hosmer–Lemeshow test.

#### Risk of Bias and Applicability Assessment of the Included External Validation Studies

**FIGURE 4:**
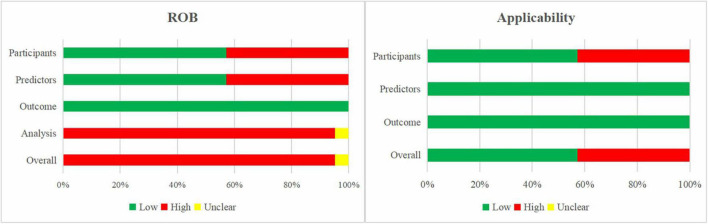
Risk of bias and applicability assessment of externally validated models using Prediction model Risk Of Bias ASsessment Tool (PROBAST).

Figure 4 presents the summary of the ROB and applicability by domain. Outcome-related ROB was low across all models. For the domain analysis, 20 models (96%) were assessed as high ROB due to inappropriate handling of missing data and inefficient presentation of calibration, while one model was assessed as unclear. This resulted in an overall high ROB for the validation of 20 models (95%) and overall unclear ROB for one model (5%).

The models’ applicability to our research question was high concern in 43% of the models, mainly due to the inclusion of participants different from those in our research question.

## Discussion

In the present systematic review, we summarize all prognostic models for developing BPD in preterm infants born at ≤32 weeks and/or ≤1,500 g birth weight. In total, 13 studies describing model development and 10 studies describing external validation were included. High ROB was observed across nearly all models, mostly due to inappropriate analysis, particularly for the handling of missing data, presenting insufficient performance statistics, and small sample size. Furthermore, several studies did not report full models, making external validation and implementation in clinical practice difficult. Meta-analysis was not possible because external validation studies of the same model were insufficient.

Prediction models are developed to support medical decision-making. Therefore, it is vital to identify a target population in which predictions serve a clinical need. Then, a representative dataset on which the prediction model is based can be developed and validated (32). In the present review, studies involving preterm infants born at ≤32 weeks and/or ≤1,500 g birth weight were included, while those that included more mature preterm infants were excluded. We excluded such studies because BPD incidence is very uncommon in infants born with birth weights of >1,500 g and after 32 weeks’ gestation (33). Accordingly, there is little clinical need for predicting BPD in such infants. Therefore, we recommend that future studies of BPD prediction models involve very low-birth weight infants or very preterm infants rather than all preterm infants.

In the present review, the outcome to be predicted was BPD. The included studies used different definitions of BPD. Most of the included studies used BPD36 as the outcome to be predicted while a smaller proportion used BPD28. Even when the scope of BPD was the same, the definitions of BPD could still differ based on the mode of respiratory support. The lack of a uniform definition of BPD in the included studies reflects the changing BPD definition of these years (34–37). Among the included studies, seven studies used the outcome BPD28, four of which used the definition proposed by the NIH in 2001 (36), and the other three only stated BPD28 as oxygen was still required at 28 days of life, and no further details were elaborated. Death is a competing outcome of BPD, some studies used the composite outcome “BPD or death” when developing prediction models for BPD. This composite outcome avoided exclusion of deceased patients who might developed BPD if they survived. Nonetheless, not all patients with early death will develop BPD. When models developed for prediction of “BPD or death” are used to predict BPD risk only, the predictive power will be lower (9), leading to a reduction in the accuracy of the prediction results. Besides, many models for prediction of death have been developed and most of them show good predictive performance (38). Utilize different models to predict BPD and death in clinical practice will probably result in higher accuracy. Therefore, BPD was selected as the outcome in our review, rather than “BPD or death.”

Most prediction models used clinical indicators including prenatal, perinatal, and postnatal factors to develop BPD prediction models. Though a large number of studies tried to explore the correlation between biomarkers and BPD, few biomarkers were included in prediction models. Of the studies included in this systematic review, only two studies constructed prediction models with biomarkers, including interleukin-6, clara cell protein-16, and Krebs von den Lungen-6 (25, 26). Genome-wide association studies and candidate gene studies investigating the correlation between genetic predisposition and BPD have been reported, but the results of different population studies are inconsistent (39). The genes specific for BPD remain to be further investigated before they could be applied to predict risk of BPD.

Similar to other systematic reviews of prediction models (38, 40, 41), we too observed several methodological shortcomings in most of the included studies.

First, although many of the studies used a large sample acquired from registries, around half of them used a sample that was too small. For example, six models were developed with samples of EPV <10, and 12 models were validated with samples with <100 events. When developing prediction models for binary outcomes, an EPV of at least 10 has been widely adopted as a criterion to minimize overfitting (42). For external validation studies, a minimum of 100 event patients is recommended (43). Recently, Riley et al. proposed formulae for calculating the minimum sample size required for developing regression-based prediction models (44), and Pavlou et al. have proposed equations for estimating the required sample size for external validation of risk models for binary outcomes (45), rendering sample size calculation more precise and efficient. Therefore, small samples should be avoided for prediction model development and validation, and it would be better to calculate the sample size with these recently reported formulae.

Second, none of the included studies handled missing data appropriately. Most did not report missing data or only included complete cases for analysis. Missing data are a common but easily underappreciated problem in prediction studies; complete case analysis can lead to biased predictor–outcome associations and biased model performance (46–48). To avoid biased model performance as a result of the deletion or single imputation of participants’ missing data, multiple imputation is recommended (46, 49–51).

Third, around 30% of the included studies describing prediction model development selected predictors *via* univariable analysis. However, univariable analysis can lead to the omission of important predictors, as selection is based on their statistical significance as a single predictor rather than in context with other predictors (52). Therefore, univariable analysis should be avoided in predictor selection. Alternative approaches include listing a limited number of candidate predictors to consider for the prediction model, and some statistical selection methods, including backward elimination and forward selection (53).

Fourth, most studies did not present discrimination and calibration simultaneously. Discrimination refers to the ability of the prediction model to separate individuals with and without the outcome event while calibration reflects the level of agreement between the observed outcomes and predictions (53). Both model discrimination and calibration must be evaluated to fully assess the predictive performance of a model. Most models in our review had a C statistic of >0.75. However, these models can still perform poorly in a new population because they could have been overfitted to the development data. Calibration was reported in around 20% of models. Nevertheless, only one study used the recommended method calibration plot, while most of the studies used only the Hosmer–Lemeshow test, which has been considered insufficient (11). Therefore, both discrimination and calibration should be reported for a model and a calibration plot is recommended for assessing calibration.

Finally, over half of the developed models were not validated internally. Internal validation is important for quantifying overfitting of the developed model and optimism in its predictive performance, except when the sample size and EPV are extremely large (11). In the present review, the most frequently used method of internal validation was split sampling, followed by bootstrapping. However, split sampling is not recommended, as it is statistically inefficient because not all available data are used for producing the prediction model (54). Bootstrapping is preferred especially when the development sample is relatively small and/or a high number of candidate predictors is studied (55).

No systematic review has been published since the systematic review of BPD prediction models in 2013 by Onland et al. (9). Compared with their review, ours has several improvements. We have followed the CHARMS checklist, and extracted and assessed most key items within 11 domains. Furthermore, we assessed the ROB and applicability of the included models with a standard tool, PROBAST.

The limitations of this review are: the exclusion of LUS-related studies. However, a meta-analysis in press has revealed that the LUS is accurate for early prediction of BPD and moderate-to-severe BPD in an average population of preterm infants of <32 weeks’ gestation (56). Second, we excluded studies that included preterm infants born at >32 weeks and >1,500 g birth weight. Therefore, studies that included very low-birth weight or very preterm infants were also excluded. Third, we excluded studies intended at predicting “BPD or death.” Therefore, we were unable to assess models for that composite outcome.

Recommendations for future development studies include collecting data by conducting prospective longitudinal cohort studies, selecting preterm infants born at ≤32 weeks and/or ≤1,500 g birth weight as participants, using the outcome definition proposed by Jensen et al. (34), choosing appropriate clinical indicators and biomarkers as predictors, using sufficiently large sample size (EPV ≥ 20) and handling missing data with multiple imputation.

## Conclusion

In this review, we included 18 studies that developed or externally validated BPD prediction models. The included studies were assessed thoroughly using the CHARMS checklist (10) and PROBAST. There were many reporting or methodological shortcomings in the included studies. For better reporting of BPD prediction models, we recommend using sufficiently large samples for developing or validating a model, using multiple imputation to address missing data, avoiding univariable analysis for selecting predictors, assessing a model’s predictive performance with both discrimination and calibration, and using internal validation for newly developed models.

## Data Availability Statement

The original contributions presented in the study are included in the article/Supplementary Material, further inquiries can be directed to the corresponding author.

## Author Contributions

H-BP, Y-LZ, and Z-BY designed the work. H-BP, Y-LZ, and YC extracted the data. Y-LZ, Z-CJ, FL, BW, and Z-BY analyzed the data. H-BP wrote the manuscript. YC and Z-BY supervised the work. All authors critically revised and approved the final version of the manuscript.

## Conflict of Interest

The authors declare that the research was conducted in the absence of any commercial or financial relationships that could be construed as a potential conflict of interest.

## Publisher’s Note

All claims expressed in this article are solely those of the authors and do not necessarily represent those of their affiliated organizations, or those of the publisher, the editors and the reviewers. Any product that may be evaluated in this article, or claim that may be made by its manufacturer, is not guaranteed or endorsed by the publisher.
