# Supplementary material for: Prediction Models for Bronchopulmonary Dysplasia in Preterm Infants: A Systematic Review
Source: Front Pediatr. 2022 May 12;10:856159. doi: 10.3389/fped.2022.856159 (PMC9133667; doi:10.3389/fped.2022.856159)
Supplement: Supplementary file 1 [file Data_Sheet_1.DOCX]

**Search strategies for PubMed (MEDLINE), Embase, and the Cochrane Library**

| **No.** | **Search terms** |
| --- | --- |
| **Pubmed(MEDLINE)** | |
| #1 | Bronchopulmonary dysplasia [MeSH] |
| #2 | Bronchopulmonary dysplasia [Title/Abstract] |
| #3 | Chronic lung disease [Title/Abstract] |
| #4 | #1 or #2 or #3 |
| #5 | (((infant OR infants OR infantile OR infancy OR newborn* OR “new born” [Title/Abstract] OR “new borns” [Title/Abstract] OR “newly born” [Title/Abstract] OR neonat* OR baby* OR babies OR premature OR prematures OR prematurity OR preterm OR preterms OR “pre term” [Title/Abstract] OR premies OR “low birth weight” [Title/Abstract] OR “low birthweight” [Title/Abstract] OR VLBW OR LBW OR ELBW))) |
| #6 | (((“predict” OR “prediction” OR “predictive” OR “predicted” OR “prognosis” OR “prognostic factor” OR “evaluation” OR “evaluation study” OR “risk factor” OR “risk assessment” OR “regression analysis” OR “logistic model” OR “statistical model” OR “algorithm” OR “multivariate analysis” OR "predictive value of tests” OR “Area Under Curve” OR "Receiver Operator Curve”))) |
| #7 | #4 and #5 and #6 |
| #8 | #7 NOT (animals [mh] NOT humans [mh]) |
| **Embase** | |
| #1 | 'lung dysplasia'/exp |
| #2 | 'bronchopulmonary dysplasia':ti,ab |
| #3 | 'chronic lung disease':ti,ab |
| #4 | #1 OR #2 OR #3 |
| #5 | infant:ti,ab OR infants:ti,ab OR infantile:ti,ab OR infancy:ti,ab OR newborn*:ti,ab OR 'new born':ti,ab OR 'new borns':ti,ab OR 'newly born':ti,ab OR neonat*:ti,ab OR baby*:ti,ab OR babies:ti,ab OR premature:ti,ab OR prematures:ti,ab OR prematurity:ti,ab OR preterm:ti,ab OR preterms:ti,ab OR 'pre term':ti,ab OR premies:ti,ab OR 'low birth weight':ti,ab OR 'low birthweight':ti,ab OR vlbw:ti,ab OR lbw:ti,ab OR elbw:ti,ab |
| #6 | 'predict' OR 'prediction' OR 'predictive' OR 'predicted' OR 'prognosis' OR 'prognostic factor' OR 'evaluation' OR 'evaluation study' OR 'risk factor' OR 'risk assessment' OR 'regression analysis' OR 'logistic model' OR 'statistical model' OR 'algorithm' OR 'multivariate analysis' OR 'predictive value of tests' OR 'area under curve' OR 'receiver operator curve' |
| #7 | #4 AND #5 AND #6 |
| #8 | human NOT animal |
| #9 | #7 AND #8 |
| **Cochrane Library** | |
| #1 | MeSH descriptor: [Bronchopulmonary Dysplasia] explode all trees |
| #2 | (bronchopulmonary dysplasia):ti,ab |
| #3 | (chronic lung disease):ti,ab |
| #4 | #1 or #2 or #3 |
| #5 | (((infant OR infants OR infantile OR infancy OR newbORn* OR "new born" OR "new borns" OR "newly born" OR neonat* OR baby* OR babies OR premature OR prematures OR prematurity OR preterm OR preterms OR "pre term" OR premies OR "low birth weight" OR "low birthweight" OR VLBW OR LBW OR ELBW))):ti,ab |
| #6 | ((("predict" OR "prediction" OR "predictive" OR "predicted" OR "prognosis" OR "prognostic factor" OR "evaluation" OR " evaluation study" OR "risk factor" OR "risk assessment" OR "regression analysis" OR "logistic model" OR "statistical model" OR "algorithm" OR "multivariate analysis" OR "predictive value of tests" OR "Area Under Curve" OR "Receiver Operator Curve"))) |
| #7 | #4 and #5 and #6 |
